# The transcriptomic profiling of SARS-CoV-2 compared to SARS, MERS, EBOV, and H1N1

**DOI:** 10.1371/journal.pone.0243270

**Published:** 2020-12-10

**Authors:** Alsamman M. Alsamman, Hatem Zayed

**Affiliations:** 1 Department of Genome Mapping, Molecular Genetics and Genome Mapping Laboratory, Agricultural Genetic Engineering Research Institute, Giza, Egypt; 2 Department of Biomedical Sciences College of Health Sciences, QU Health, Qatar University, Doha, Qatar; University Hospital Tuebingen, GERMANY

## Abstract

The SARS-CoV-2 (COVID-19) pandemic is a global crisis that threatens our way of life. As of November 18, 2020, SARS-CoV-2 has claimed more than 1,342,709 lives, with a global mortality rate of ~2.4% and a recovery rate of ~69.6%. Understanding the interaction of cellular targets with the SARS-CoV-2 infection is crucial for therapeutic development. Therefore, the aim of this study was to perform a comparative analysis of transcriptomic signatures of infection of SARS-CoV-2 compared to other respiratory viruses (EBOV, H1N1, MERS-CoV, and SARS-CoV), to determine a unique anti-SARS-CoV-2 gene signature. We identified for the first time that molecular pathways for heparin-binding, RAGE, miRNA, and PLA2 inhibitors were associated with SARS-CoV-2 infection. The *NRCAM* and *SAA2* genes, which are involved in severe inflammatory responses, and the *FGF1* and *FOXO1* genes, which are associated with immune regulation, were found to be associated with the cellular gene response to SARS-CoV-2 infection. Moreover, several cytokines, most significantly *IL-8* and *IL-6*, demonstrated key associations with SARS-CoV-2 infection. Interestingly, the only response gene that was shared among the five viral infections was *SERPINB1*. The protein-protein interaction (PPI) analysis shed light on genes with high interaction activity that SARS-CoV-2 shares with other viral infections. The findings showed that the genetic pathways associated with rheumatoid arthritis, the AGE-RAGE signaling system, malaria, hepatitis B, and influenza A were of high significance. We found that the virogenomic transcriptome of infection, gene modulation of host antiviral responses, and GO terms of SARS-CoV-2 and EBOV were more similar than to SARS, H1N1, and MERS. This work compares the virogenomic signatures of highly pathogenic viruses and provides valid targets for potential therapy against SARS-CoV-2.

## Introduction

COVID-19 is caused by severe acute respiratory syndrome coronavirus 2 (SARS-CoV-2). As of November 18, 2020, the SARS-CoV-2 pandemic has spread to more than 213 countries and territories with approximately 55.9 million confirmed cases and ~ 2.4% mortality [1, 2]. Humans have experienced several novel viral outbreaks, such as Ebola virus disease (EBOV) and H1N1, in 2009 and 2013–2016, respectively. The main reservoir for EBOV is considered to be bats where the magnitude of its outbreak was unprecedented, with > 28 500 reported cases and > 11 000 deaths in West Africa [3]. On the other hand, swine H1N1 spread rapidly throughout the world, leading the WHO to declare it a pandemic on June 11, 2009 [4]. A typical biological response to different viral infections could be identified, where some particular genes are dysregulated during an infection by specific viruses. Such responses may have a major impact on the ability of the host to mount an adaptive host response. For instance, both MERS and SARS-CoV induce a similar pattern of activation of recognition receptors and the interleukin 17 (IL-17) pathway [5].

We compared the transcriptomic data of SAR-CoV-2 to that of MERS-CoV, SARS-CoV, H1N1, and EBOV. We focused our analysis on viral infections that are evolutionarily related to SAR-CoV-2 (SARS-CoV, MERS-CoV), have the same aggressiveness (EBOV), or attack the same human organs (MERS-CoV, SARS-CoV, H1N1, EBOV). We identified common and specific differentially expressed genes in the response to SARS-CoV-2 that are shared with SARS-CoV, MERS-CoV, H1N1, and EBOV. We performed chromosomal location, gene ontology and protein-protein interactions analyses for these genes in order to understand SARS-CoV-2’s unusually high infection and mortality rates.

## Material and methods

### Datasets

The gene expression data of SARS-CoV-2, EBOV, H1N1, MERS-CoV, and SARS-CoV were retrieved from the NCBI-GEO archive [6], with ID GSE147507, GSE86539, GSE21802, GSE100504, and GSE17400, respectively. These data are based on Affymetrix human genome gene chip sets and Illumina NextSeq 500, revealing the gene expression profiles of *in vitro* and *in vivo* infections (**S1 Table**).

### Data normalization and filtration

**Fig 1:**
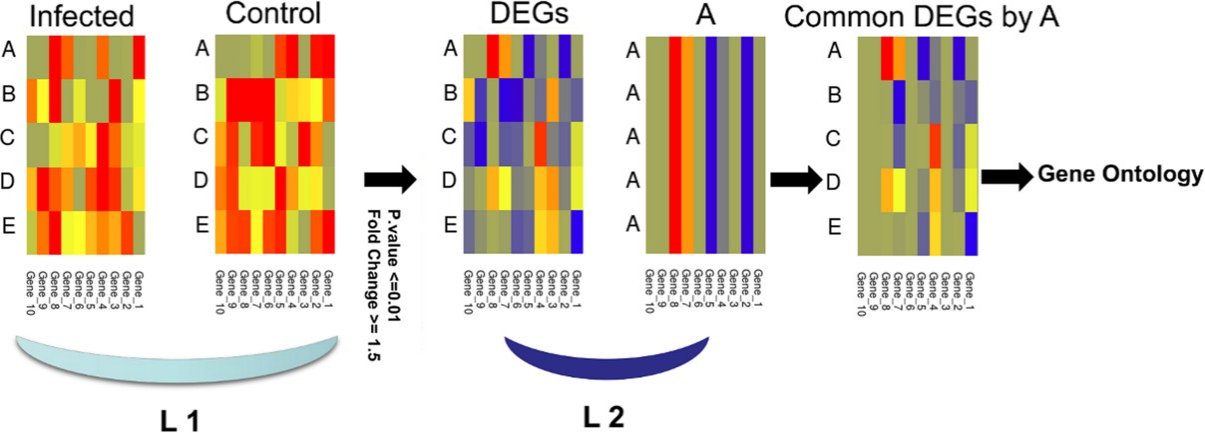
Data filtration and analysis protocol followed to study SARS-CoV-2 (A), EBOV (B), H1N1 (C), MERS-CoV (D), and SARS-CoV (E) data sets. The first level of filtration (L1) was conducted using control samples, significance and fold change, while the second level of filtration (L2) was done by removing genes that are not expressed in host response to SARS-CoV-2.

Due to the difficulty of finding different data that are produced using a common cell line infected by the five viruses studied, we have adopted a specific procedure to focus on DEGs, which reflects a variation in the response of the host to the type of virus rather than the experimental conditions. Data analysis for all viral infections used was conducted on a stand-alone basis, where control/reference samples were used as a baseline for each experiment. This step should decrease background gene expression and illuminate those related to host response to infection. Additionally, we focused on the analysis of induced genes that are common between SARS-CoV-2 and the other four viral infections, discarding genes that are not expressed in SARS-CoV-2, which adds additional filter layer. Using control samples, and focusing on these common genes were used to encounter the possible gene expression differences specific to each cell type (**Fig 1**).

### Data analysis

The identification of the differentially expressed genes (DEGs) in the transcription profile was performed with the GEO2R tool [7] and differential expression analysis using DESeq2 and DEApp (fold change ≥ 1.5 and FDR adjusted p-value ≤0.01) [8] using default parameters. All transcriptomic profiles consist of control cell and infected cells, where the control cells were used as the baseline for DEGs analysis, using the default setting of all programs mentioned in the Methods section. To avoid expression profiles linked to cell types or infection conditions and to shed more light on gene expression that reveals a unique SARS-CoV-2 signature of the host response, we excluded all genes that were not expressed in response to SARS-CoV-2, focusing on genes that were shared between the gene profiles of SARS-CoV-2 and the other viruses. The DEGs were characterized for each sample (p-value ≤ 0.01) and were used as queries to search for enriched biological processes. The Gplot package in R was used to construct the gene expression heatmaps. The evaluation of the protein interactions and gene ontology (GO) enrichment was conducted with the STRING database [9]. Cytoscape software was used to visualize the structures of the protein-protein interaction (PPI) networks [10]. Circos software and GeneSyno [11, 12] was used to represent gene expression and gene ontology analysis of the host response to viral infections based on human genome data (GRCh38). The online tool Draw Venn Diagram (http:/bioinformatics.psb.ugent.be/webtools/Venn/) was used to sketch a Venn diagram demonstrating some analysis information. PERL Python and R programming language scripts used to perform these analyzes are freely available at https://github.com/AlsammanAlsamman/Alsamman-and-Zayed-SARS-CoV-2.

## Results

**Fig 2:**
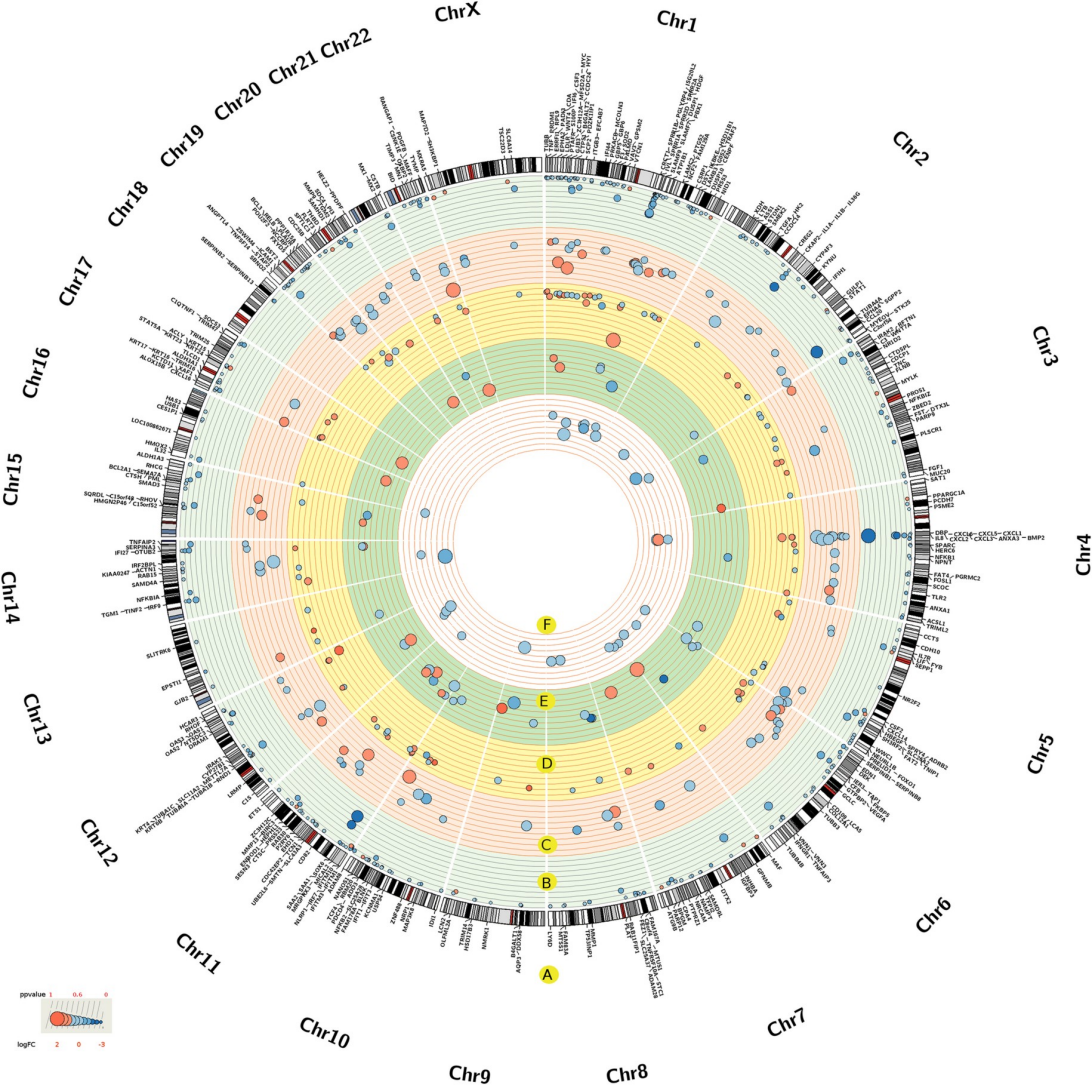
Significant DEGs across the five transcriptomic profiles, corresponding genes, chromosome locations, gene expression and significance scores. The DEGs related genes and chromosomal locations (A). The DEGs information regarding the host response to SARS-CoV-2 (B), EBOV (C), MERS-CoV (D), H1N1 (E) and SARS-CoV (F) viral infections. The p-values were scaled across gene profiles according to maximum and minimum values. The sizes and colors of the circles respectively indicate the significance and gene expression (LogFC) scores of the DEGs.

We investigated the unique transcriptomic gene expression signature that was induced by the infection of SARS-CoV-2 (GSE147507) compared to EBOV (GSE86539), H1N1 (GSE21802), MERS-CoV (GSE100504), and SARS-CoV (GSE17400). DEGs were investigated in each profile. The chromosome locations of these DEG sets are categorized according to the viral infection in **Fig 2**, and their significant involvement in the infection response according to the p-values is visualized in **Fig 2A–2F** and **S2 Table**.

### Transcriptional features of SARS-CoV-2 infection

**Fig 3:**
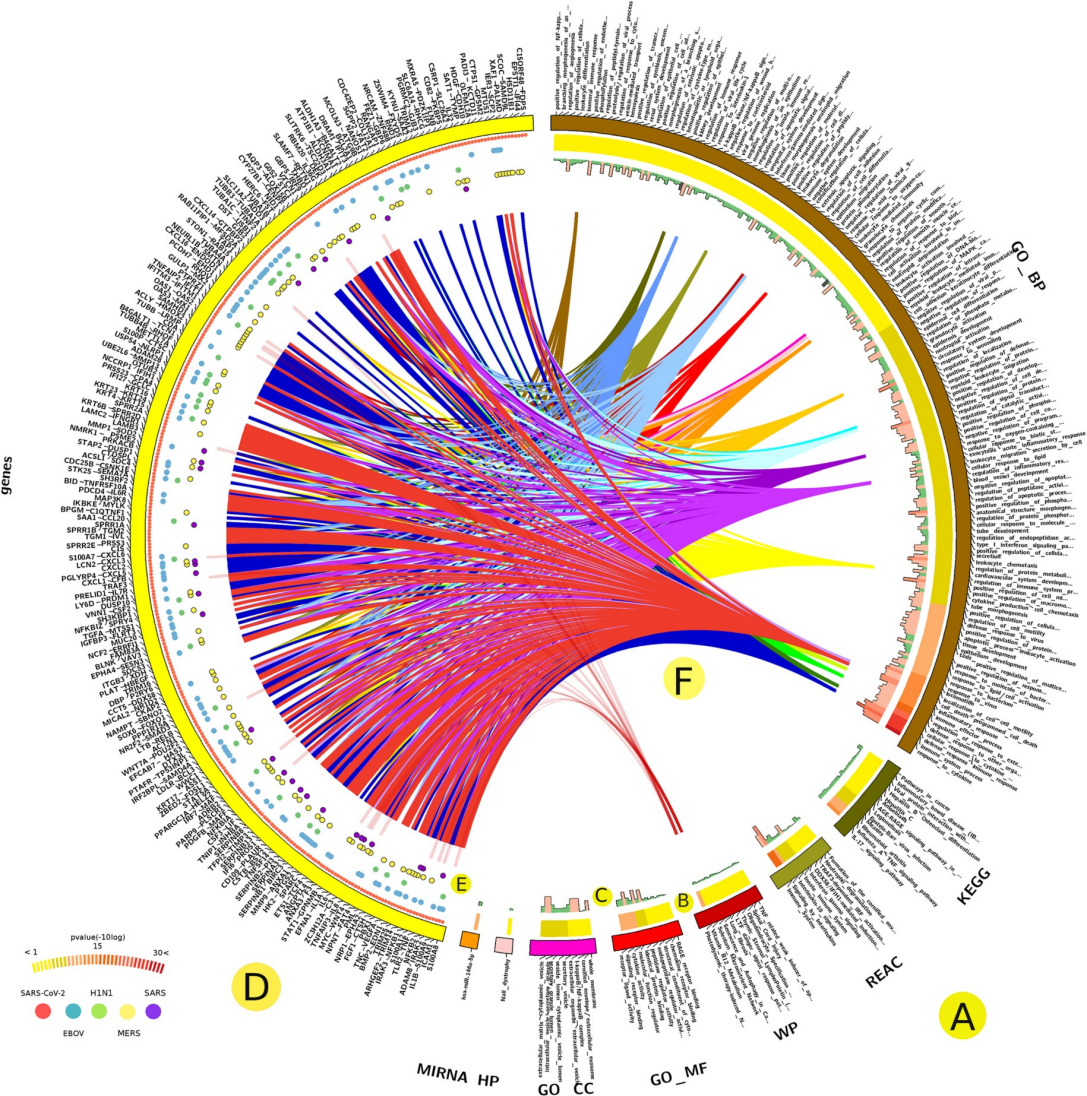
Analysis of the gene enrichment of DEGs correlated with the host response to SARS-CoV-2. Categories of GO terms (A), significance scores (-10logp-value) (B), and number of associated DEGs (C). The SARS-CoV-2-associated DEGs (D), status across the studied infectious diseases (E), and selected linked GO terms (F).

We identified 358 DEGs with a significant associated p-value < 0.01 to SARS-CoV-2. Of these, *SAA2*, *CCL20*, and *IL8* were highly significant (**Fig 2B and S2 Table**). The analysis of gene enrichment of DEGs associated with the host response to SARS-CoV-2 highlighted several GO terms (**Fig 3**), including leukocyte activation, humoral immunity, myeloid cell activation, neutrophil activation, tuberculosis response, and miRNA involvement in the immune response. Additionally, GO terms that are correlated with cell death were highly and consistently regulated in all viral infections (**Fig 3C and 3E**). GO cytokine response terms, IL-17 signaling pathway, NF-kB signaling, TNF signaling pathway, and NF-kappa B signaling were among the most significant pathways associated with SARS-CoV-2 (**Fig 3B**).

**Fig 4:**
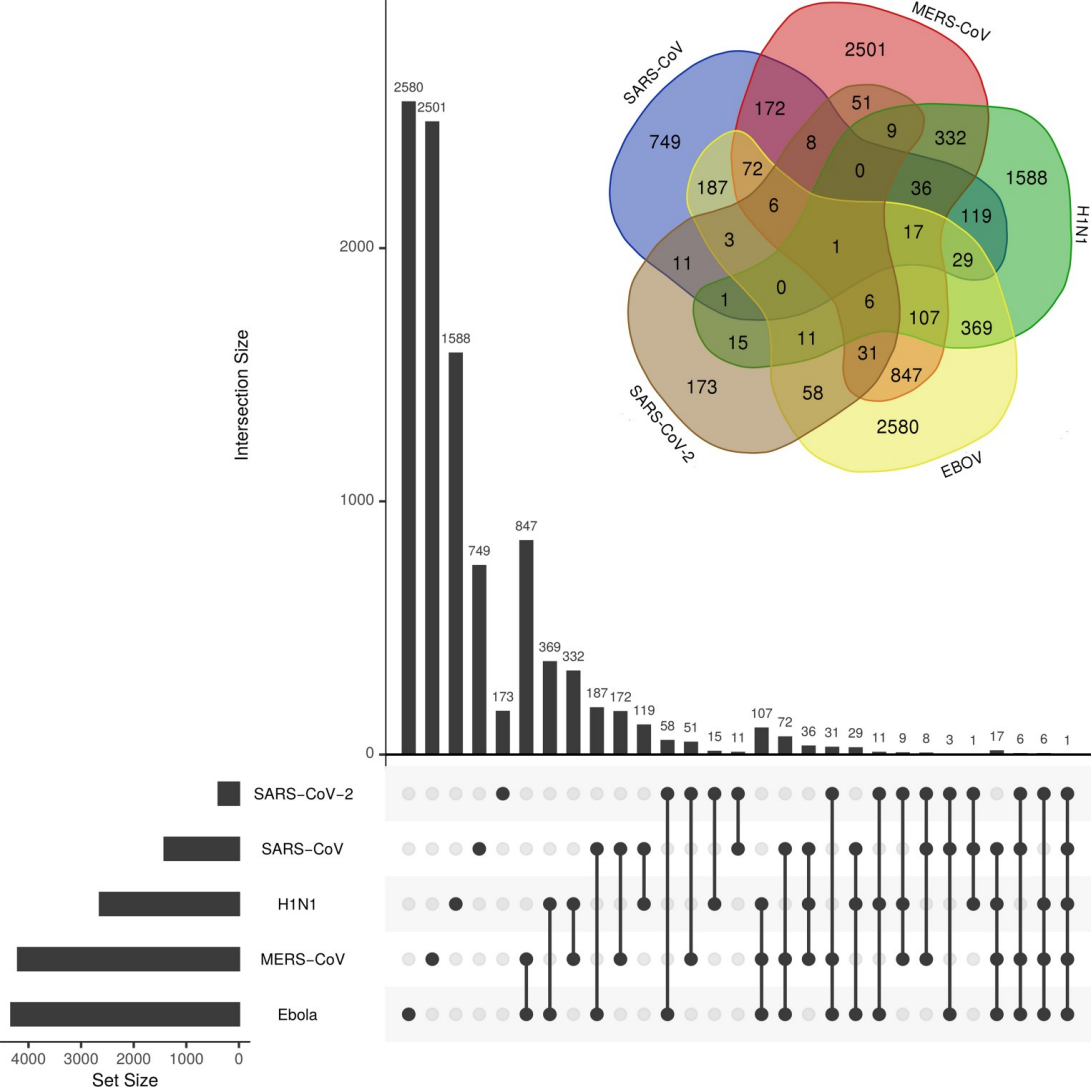
The Venn diagram of viral-associated genes. The number of uniquely shared genes associated with the host response to SARS-CoV-2, EBOV, H1N1, MERS-CoV, and SARS-CoV viral infections.

We particularly focused on the analysis of DEGs induced during SARS-CoV-2 infection and its overlap with the other four viral infections. We found 173 DEGs were unique to SARS-CoV-2 (**Fig 4 and S3 Table**). Of these genes, *SAA2* was the most significant (-10logp-value of 81) (**S2 Table**); the distinctive genes in the SARS-CoV-2’s infection response were *CSF3*, *CSF2*, *IL1B*, and *PTGS2* (**S2 Table**). GO analysis demonstrated that these genes were linked to the IL-17 signaling pathway and were induced as a response to Rhinovirus infection (**S1 Fig**). Overall, the biologic process terms, such as keratinocyte/epithelial cell differentiation, organ development, cell component movement and cell death, were very significant among these genes (**S4 Table**), and molecular functions such as RAGE receptor binding, cytokine activity, and metal ion binding were also highly recognized (**S4 Table**).

### Transcriptional features SARS-CoV-2 shares with the other studied viral infections

Comparative gene expression analysis of the five viral infections (SARS-CoV-2, EBOV, H1N1, MERS-CoV, and SARS-CoV) yielded *SERPINB1* as the common response gene among the five infections. SARS-CoV-2 and EBOV shared 58 DEGs, including *TNIP1*, *ICAM1*, and *CFB* that were highly significant (-10logp-value > 40), while genes such as *TLR2*, *FOXO1*, and *MYC* were highly associated with cytokine response and cell death (**S2 Fig** and **S2 Table**). The GO molecular terms of these genes highlighted the biological functions of phospholipase inhibitor activity (including phospholipase A2) and heparin binding (including glycosaminoglycan). While biological processes such as cell surface receptor signaling pathways and cell death were significantly represented by a large number of genes (**S4 Table**). The MERS-CoV-shared genes *KRT6B* and *TNFAIP3* had a high p-value associated with SARS-CoV-2, whereas genes such as *OAS1-3*, *IRF9*, *IRF7*, *STAT1*, *PML* and *IFIH1* were highly associated with host responses to viral infections and type I interferon (**S3 Fig**). Biological processes related to virus response, Type I interferon signaling and the cytokine-mediated signaling pathway were highly redundant, while the biological functions of 2-5-oligoadenylate synthetase activity, double-stranded RNA binding, adenyltransferase activity, metal ion binding and related to growth activity, such as epidermal growth, were quite significant (**S4 Table**).

SARS-CoV-2, EBOV, and MERS-CoV share uniquely 31 genes, of which *BIRC3*, *MX1*, and *IL8* are strongly linked to SARS-CoV-2 (-logp-value 23, 37, and 105, respectively) (**Fig 4 and S2 and S3 Tables**). Among these genes, *DDX58* and *IFIT1* are highly associated with cytokine response, the NF-kappa B signaling pathway, and immune responses to virus infection (**S4 Fig**). On the other hand, SARS-CoV-2 and SARS-CoV shared 30 genes (**S3 Table**), of which 11 genes were unique to both viruses (**Fig 4**). These genes are involved in immune system regulation, some of which are associated with the host response to rheumatoid arthritis (*CCL20*, *IL1A*, and *MMP1*). *CCL20* and *INHBA* were significantly induced by SARS-CoV-2 infection (10logp-value of > 40). Interestingly, genes related to vitamin D regulation (*CYP27B1*), inflammation (*IRAK3*) and pulmonary fibrosis (MMP1) were significantly induced by both viruses. The GO analysis showed that four GO terms were uniquely shared between SARS-CoV-2 and SARS-CoV (**S3 Table**) and also with legionellosis and amoebiasis infection. The intersection of DEGs between SARS-CoV-2 and H1N1 showed 15 genes were uniquely shared (**S3 Table**). Some of these genes were associated with neutrophil degranulation (*B4GALT1*, *VNN1*, *LCN2*, *CSTB*, and *CTSC*). *SAA1* was highly associated with SARS-CoV-2 (10logp-value = 55) (**S2 Table**), and it has been reported to be involved in the host response to amyloidosis and rheumatoid arthritis [13]. In addition, 3 GO terms were uniquely shared between the two virus infections (GO:0031983, GO:0034774, and GO:0060205), which were mainly linked to the lumen vesicles. Interestingly, SARS-CoV-2, H1N1, and SARS-CoV shared overexpression of *MAF* compared with EBOV and MERS-CoV. The *MAF* transcription factor is a key component in the immune response to several diseases, regulating disease-specific gene networks [14].

The gene expression profile of SARS-CoV-2 highlighted genes such as *MX1*, *BIRC3*, *IRAK2*, *CXCL5*, *NRCAM*, *FGF1*, *MMP9*, *SAA1*, *LCN2*, *IFI27*, *TNFAIP3*, *OAS1*, *IL6*, *XAF1*, *IL8*, and *CXCL3* compared to EBOV, H1N1, MERS-CoV, and SARS-CoV. The host gene expression of these genes changed exponentially relative to other viral infections (**Figs 2 and S5 and S3 Table**). These genes are mostly related to the IL-17 signaling pathway, the TNF signaling pathway, and the host response against viral infection (**S6 Fig**).

**Fig 5:**
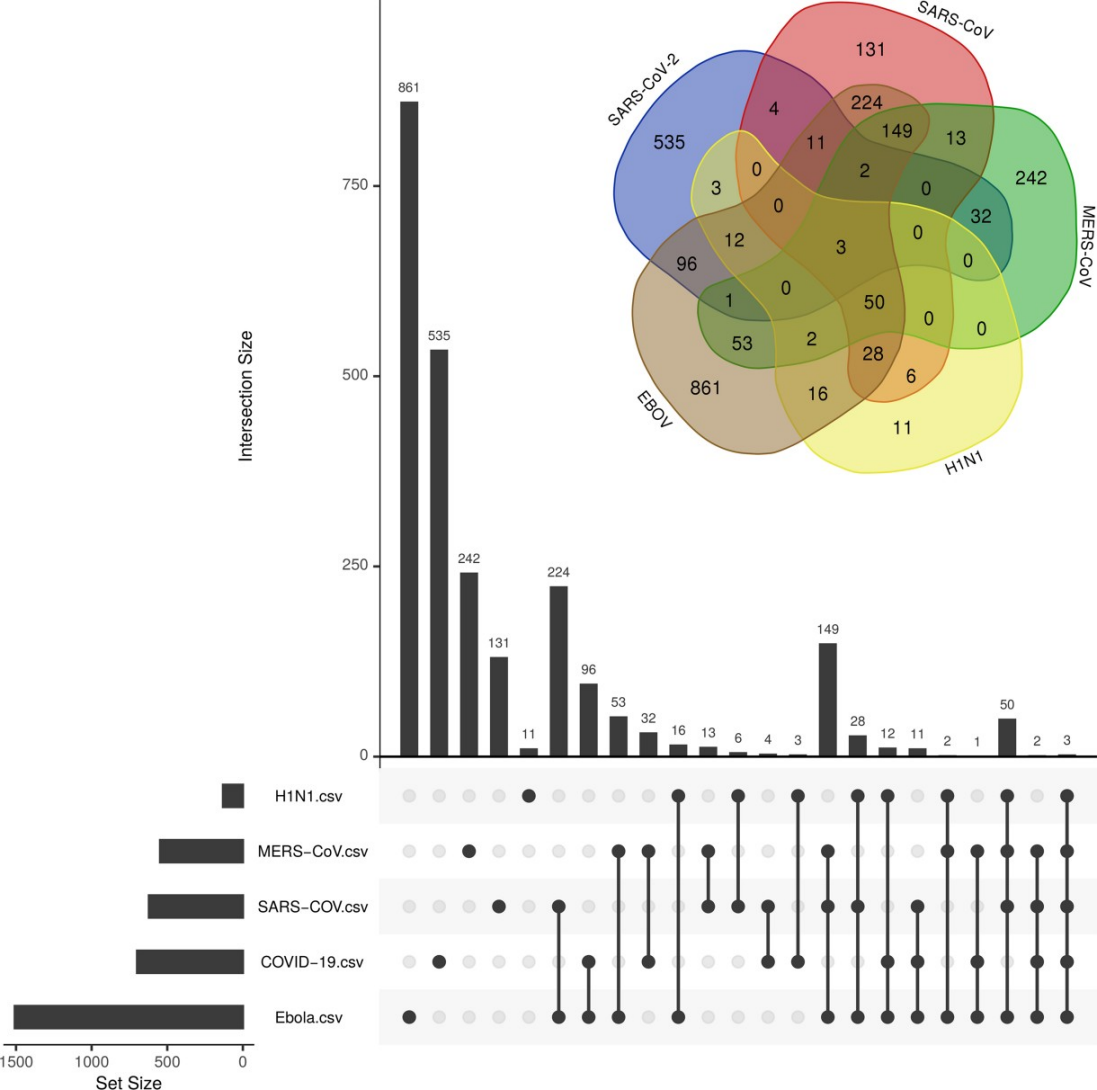
The Venn diagram of viral associated GO terms. The number of uniquely shared GO terms of DEGs associated with the host response across SARS-CoV-2, EBOV, H1N1, MERS-CoV, and SARS-CoV viral infections.

Analysis of gene enrichment showed that only three GO terms were shared between SARS-CoV-2 and the other viral infections (**Fig 5 and S3 Table**), including cellular component, protein binding and cytoplasm. SARS-CoV-2 was uniquely characterized by 535 GO terms, including stimulus response, cell communication, and defense response to bacterial infection (**S6 Table**). SARS-CoV-2 shared 96 GO terms with EBOV, where GO terms related to the regulation of cell death were substantially shared. In addition, SARS-CoV-2 and MERS-CoV uniquely shared 32 GO terms, most of which were linked to the cell defense against viral infection and immunity, and metal ion response (**Fig 5 and S3 and S6 Tables)**.

### PPIs involved in the mechanisms of infection and host response

**Fig 6:**
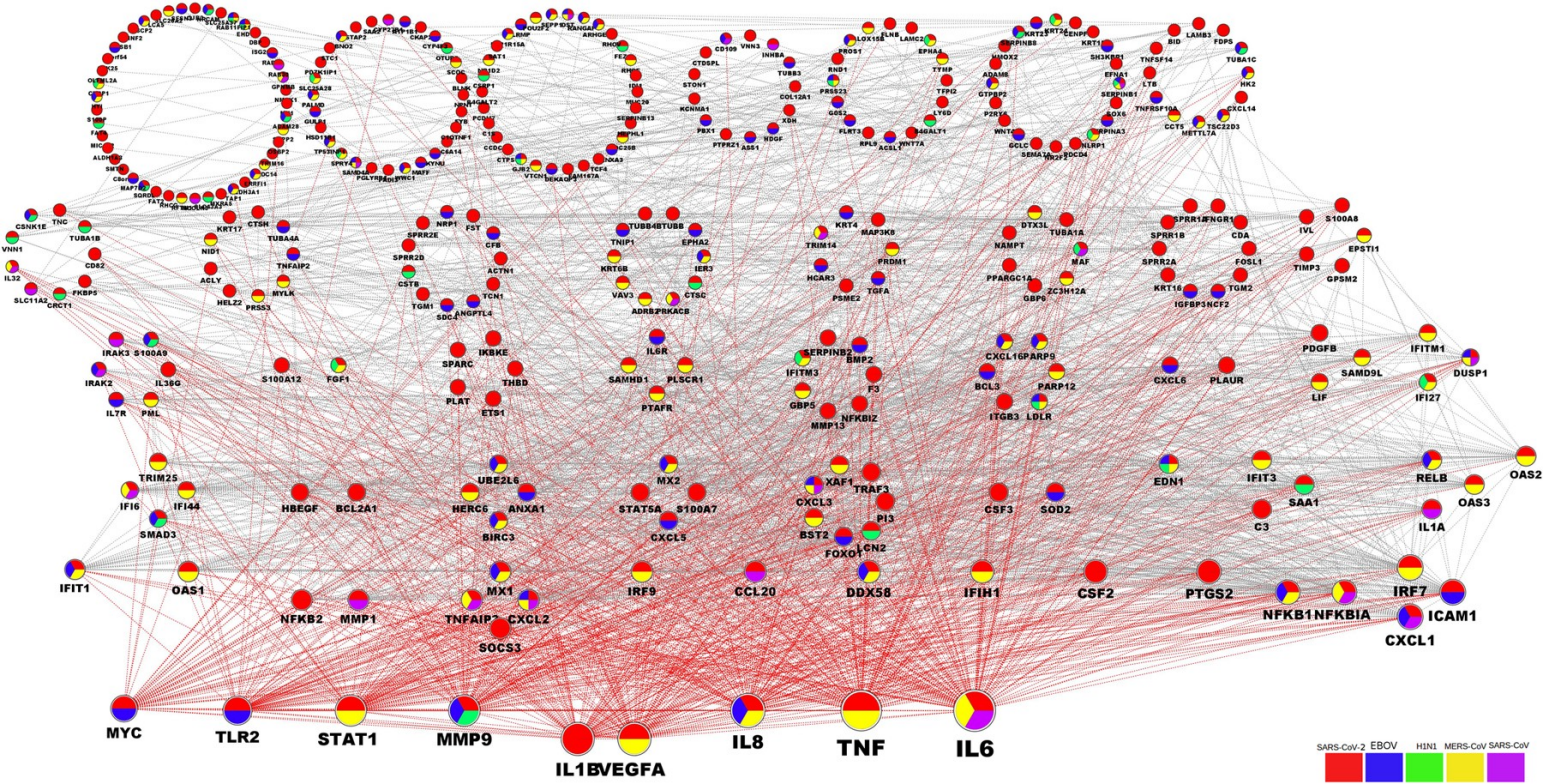
The PPIs network of DEGs associated with SARS-CoV-2. The PPI of host expressed DEGs under SARS-CoV-2 infection. DEGs shared between SARS-CoV-2 and EBOV, H1N1, MERS-CoV, and SARS-CoV are color-coded according to the kind of infection. Edges of nodes are mutual protein interactions, where the edges of high-activity nodes are red-colored. The gene node size is relative to its interaction activity. The DEGs are collected in different groups according to their level of interaction activity, where genes with an equal number of interactions are clustered into one group (circle).

**Fig 7:**
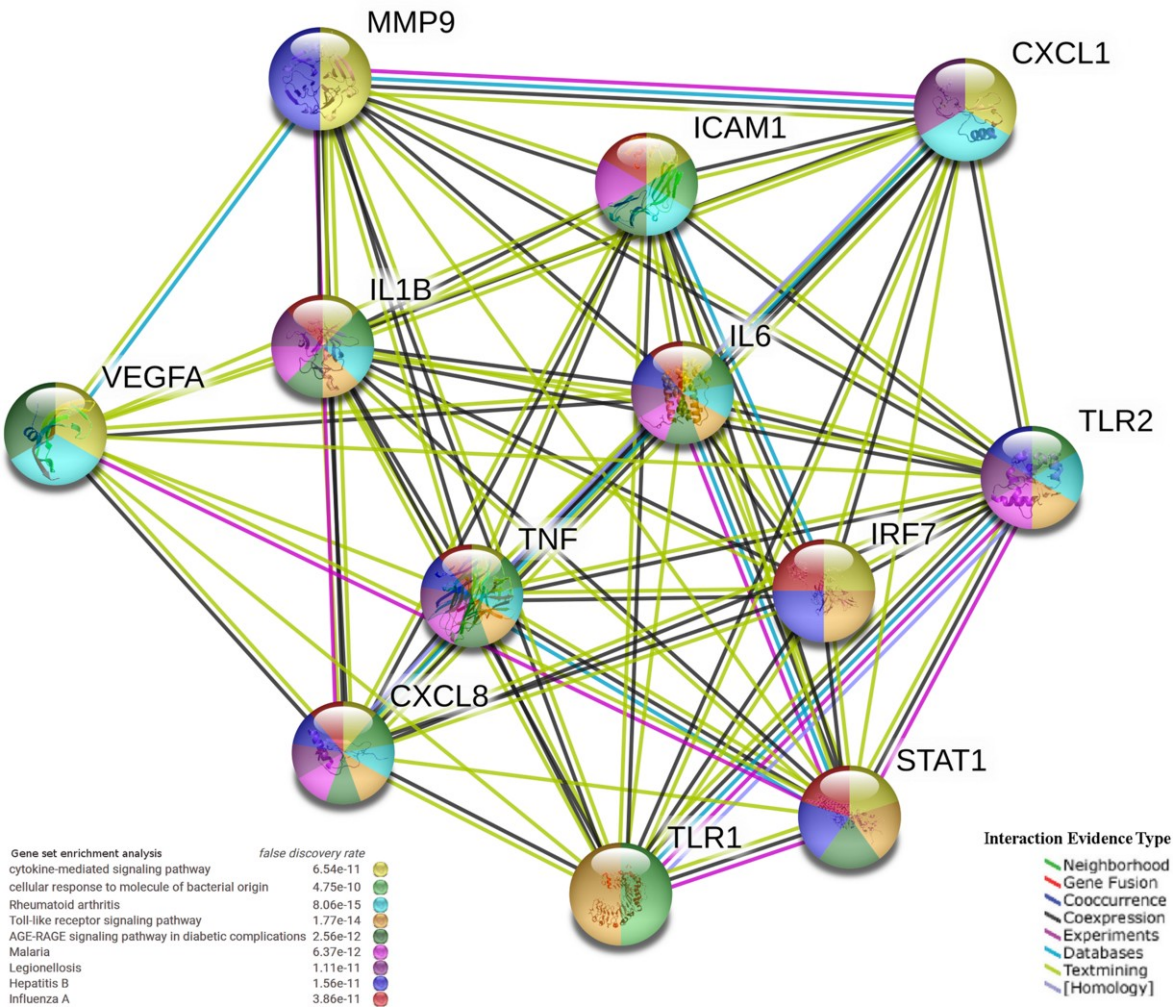
The PPIs network and gene enrichment analysis of highly interactive genes associated with SARS-CoV-2. Each node represents a protein and each edge stands for an interaction, color-coded by the type of the interaction evidence.

We used the PPI association network analysis to identify the shared DEGs between SARS-CoV-2 and the other four viral infections. Genes have been clustered into groups according to their interaction activity, where genes with an equal number of interactions are clustered into one group, where genes have been classified according to the type of infection they are involved in (**Fig 6**). The PPI network clustering highlighted genes such as *IL6*, *TNF*, *IL8*, *VEGFA*, *IL1B*, *MMP9*, *STAT1*, *TLR1*, *CXCL1*, *ICAM1*, *TLR2*, and *IRF7* with high interaction activity. Some genes were associated with both SARS-CoV-2 and EBOV, and a few were shared with MERS-CoV (**Fig 6**). The PPI analysis and gene enrichment analysis of these hyper-interactive genes showed significant biological functions connected to the AGE-RAGE signaling pathway and the host response to rheumatoid arthritis, malaria, hepatitis B, and influenza A (**Fig 7**).

## Discussion

This study mainly aimed to determine the unique host gene expression signature response to SARS-CoV-2 infection compared to SARS-CoV, MERS-CoV, EBOV, and H1N1, which will help us to understand the differences and similarities in host responses to various respiratory viruses. Although the cell lines were different in each viral infection, we studied only the DEGs associated with SARS-CoV-2 that were similar to other viral infection responses. To our knowledge, this is the first study to perform such a transcriptomic comparison among these five viral infections.

### Significant and unique transcriptional responses to SARS-CoV-2 infection

The analysis of DEGs to SARS-CoV-2 infection identified the *SAA2*, *CCL20*, and *IL8* genes as being significantly induced (**Fig 2B and S2 Table**). Recently, a link between the serum amyloid A 2 (*SAA2*) gene and SARS-CoV-2 infection has been proposed as a biomarker to differentiate the severity and prognosis of cases of SARS-CoV-2 infection [15]. In addition, we observed the uniqueness of the *SAA2* gene expression in SARS-CoV-2 infection compared to SARS-CoV, MERS-CoV, EBOV, and H1N1 viral infections (**Fig 4 and S3 Table**). Multiple genes belonging to the interleukin gene family, such as *IL6*, *CXCL1*, *3* and *5*, and *IL-17*, demonstrated significant responses to SARS-CoV-2 infection (**Fig 2 and S2 Table**). In addition, the *IL8* gene, which has been related to immune stimulus and is a recognized locus of susceptibility to a specific respiratory virus, was also induced [16]. Such genes serve as key factors for controlling the growth of endothelial cells, which is a major player in SARS-CoV-2 infection [17, 18].

GO-based gene enrichment analysis demonstrated that many involved biological processes were closely related to the immune response (**Fig 3A**), including myeloid cell activation and neutrophil activation (**Fig 3C and 3E**). Interestingly, the miRNAs-related gene pathways were overexpressed as a response to SARS-CoV-2 infection, and they are known to play an important role against viral infection [19]. Activation of miRNAs as a defense mechanism during lung infection could be related to their important roles in physiological and pathological processes in the lung [20]. Studying such processes could open a new avenue for treatment of COVID-19. We identified a strong association between SARS-CoV-2 infection and GO related to the Nuclear Factor Kappa-B (NF-kB) signaling and Tumor Necrosis Factor (TNF) signaling pathways (**Fig 3B**). The NF-kB pathway is closely related to pro-inflammatory and pro-oxidant responses, and responses in acute lung injuries, where its activation has been proposed as a potential adjuvant treatment for SARS-CoV-2 [21]. Similarly, TNF receptors are mainly involved in the inflammatory response and may have a role to play in viral pathogenesis [22].

Among the genes that are unique in the host response to SARS-CoV-2 are *CSF2/3* and *PTGS2*, which known to be involved in the immune responses against Rhinovirus infection (**S1 Fig**). The relationship between the prostaglandin-endoperoxide synthase 2 (*PTGS2*/*COX‐2*) gene and the host response to SARS-CoV-2 infection could be due to its role in down regulating NF-κB mediated transcription, which is a critical element in the replication of some viruses, such as HIV-1 [23]. Colony-stimulating granulocyte factor (*G-CSF)* can alter the function of T-cells and induces the Th2 immune response [24]. There is also some evidence of a link between an elevated G-CSF expression level and the induction of the cellular immune response in H1N1 infected individuals [25].

The GO-associated molecular function in the SARS-CoV-2 host response yielded terms such as receptor for advanced glycation end products (*RAGE*) and metal ion binding (**Fig 3B and S4 Table**). RAGE is highly expressed only in the lung and is rapidly induced at inflammatory sites, primarily in inflammatory and epithelial cells. The triggering and upregulation of RAGE by its ligands correlate with increased survival rates [26]. Additionally, RAGE has a secretory isoform that can have an independent causative effect on community-acquired pneumonia, such as pandemic influenza (H1N1) [27]. Although there is no evidence to link this to SARS-CoV-2 infection, it is worth further investigation.

### EBOV shares more, and SARS-CoV shares fewer, DEGs with SARS-CoV-2 than other viral infections

Among the five viral infections, we found that GO terms were mostly enriched between SARS-CoV-2 and EBOV (**Fig 5 and S3 Table**). Such an overlap suggested the common involvement of certain genes and gene families, which could explain the aggressiveness of SARS-CoV-2 infections. Within these GO enriched pathways, *TNIP1*, *ICAM1*, and *CFB* were most significantly associated with SARS-CoV-2 (logp-value > 40) (**Fig 2 and S2 Table)**. *TNIP1* reduction sensitizes keratinocytes to post-receptor signaling after interaction with *TLR* agonists and it has the ability to activate immune cells and induce inflammation [28]. The correlation between *TNP1* and SARS-CoV-2 (**Fig 2 and S2 Table**) could be due to its role in suppressing the NF-kB pathway and therefore regulating the overexpression of viral proteins [29, 30]. The ICAM-1 protein plays a key role in controlling viral infection in lung epithelial cells during the early stages of infection, influencing the migration of immune effector cells into the airways [31]. Forkhead Box O1 (*FOXO1*) is a transcription factor that negatively regulates the cellular antiviral response by promoting the degradation of interferon regulatory transcription factor 3 (*IRF3*) [32]. In addition, it has an intrinsic role in the post-effector memory program, which is important for the formation of long-lived memory cells capable of immune reactivation [33].

GO analysis of genes uniquely shared between SARS-CoV-2 and EBOV highlighted the activity of the inhibitors of phospholipases, in particular, phospholipase A2 (PLA2) (**Fig 5 and S4 Table**). Interestingly, synthetic and natural PLA2 inhibitors have been a viable treatment for oxidative stress and neuroinflammation associated with neuropathogenic disorders [34]. Some reports have suggested a potential link between PLA2-generated lipid mediators and viral infection, where these infections alter the lipid mediators of this pathway to initiate infection and pathogenesis [35]. Given the important association between heparin-binding GO and activation of T cells against virus infections such as influenza [36], their interaction with SARS-CoV-2 infection has not been documented. In comparison, glycosaminoglycan-binding molecules are essential for the action of certain *in vivo* chemokines. Some glycosaminoglycans are required for respiratory syncytial viral infection and are important for the entry of a bacterial pathogen into a biological system [37]. Some oncofetal antigens that target such proteins are used to control malaria parasites [38]. This might support some of the recent suggestions of using pharmaceuticals derived from glycosaminoglycan to control infection with SARS-CoV-2 [39].

MERS-CoV uniquely shared 51 DEGs with SARS-CoV-2 (**Fig 4 and S3 Table**). Among the most significant shared genes that were associated with SARS-CoV-2 are *KRT6B* and *TNFAIP3*. Keratin 6B (*KRT6B*) is a type II cytokeratin, which is an important biomarker for lung adenocarcinoma [40]. These genes are known as virus-induced host factors that control the recruitment of T-cells and correlate with chronic virus infections [41]. In addition, the tumor necrosis factor, alpha-induced protein 3 (*TNFAIP3*), is a central regulator of immunopathology and is associated with the maintenance of immune homeostasis and severe viral infections [42, 43]. We identified many DEGs that are classified as “antiviral genes” that were shared between MERS-CoV and SARS-CoV-2 (**Fig 4**). Most of these DEGs were associated with the host response to virus infection and type I interferons (**S3 Fig**), while others such as *IRF9*, *PML*, *IRF7*, *STAT1* and *IFIH1* were related to interferon signaling [44].

The low number of uniquely shared DEGs between SARS-CoV-2 and SARS-CoV and SARS-CoV-2 and H1N1 compared to SARS-CoV-2 and EBOV could be explained by the unique signature of SARS-CoV-2 and the high pathogenicity and aggressiveness that both SARS-CoV-2 and EBOV share, where the similarity of the genome and the common descent cannot be emphasized by a common biological host response. On the other hand, the uniquely shared DEGs and gene GO terms between SARS-CoV-2 and SARS-CoV-1 and SARS-CoV-2 and H1N1 have highlighted the role of vitamin D regulation (*CYP27B1*) and transcription factors (*MAF*) in immune functionality against SARS-CoV-2 [45]. Such information could explain the recent reported links between vitamin D and the response of immunity to SARS-CoV-2 infection [46]. It also highlighted the similarity of the host response to viral and bacterial infections.

### Most common transcriptional responses among the studied viral infections

The host response to the five viruses shared the plasminogen activator (*SERPINB1*) as a common gene signature (**Fig 4 and S3 Table**). This gene is highly correlated with lung chronic airway inflammation such as in asthma [47]. *SERPINB*1 acts in host-pathogenic interactions and possesses some antiviral activity across infections of rhabdovirus, hepatitis C, and influenza A [48, 49].

The SARS-CoV-2 gene expression profile demonstrated multiple genes in conjunction with EBOV, H1N1, MERS-CoV, and SARS-CoV (**Figs 4 and S6 and S5 Table**). Most of these genes were linked to the viral infection immune response of the host, except for genes such as *FGF1* and *NRCAM*. The Neuronal Cell Adhesion Molecule (*NRCAM*) is related to neurological diseases such as Alzheimer [50]. Significant *NRCAM* gene expression has been observed under specific circumstances, such as neuroinflammation triggered by influenza A long-term viral infection [51]. *FGF1*, also known as acidic fibroblast growth factor (aFGF), is a cellular growth factor and signaling protein encoded by the *FGF1* gene. *FGF1* is a strong angiogenic factor that controls the development of new blood vessels [52] and has been detected while studying endothelial cells infected with influenza virus [53].

SARS-CoV-2, EBOV, and MERS-CoV shared 31 unique genes, among which *BIRC3* and *MX1* were highly linked to SARS-CoV-2 (**Fig 4 and S3 Table**). The Baculoviral IAP Repeat Containing 3 (*BIRC3*) is associated with marginal zone B-cell lymphoma and was suggested as a novel NK cell immune checkpoint in cancer [54, 55]. While MX Dynamin Like GTPase 1 (*MX1*) is an interferon-inducible protein that is associated with viral infections by influenza and viral encephalitis [56, 57]. A link between the gene expression of *BIRC3* and *MX1* has been hypothesized as part of a small group of genes controlling the host response against viral infections, including human herpes virus type 6Α (HHV-6Α) infection [58]. Additionally, the *Mx1* protein contributes to the novel antiviral activity against classical swine fever virus [59]. Among the genes that were uniquely shared among SARS-CoV-2, EBOV, and MERS-CoV, Interferon Induced Protein With Tetratricopeptide Repeats 1 (*IFIT1*) and DExD/H-Box Helicase 58 (DDX58) had high significant potentiality (**S4 Fig**). Recently, the uniqueness of *DDX58* gene expression under SARS-CoV-2 viral infection has been reported [60]. *IFIT1* plays a crucial role in some viral infections, where hepatitis E virus polymerase binds to *IFIT1* to shield the viral RNA from translation inhibition mediated by *IFIT1* and enhances the interferon response in murine macrophage-like cells [61, 62].

### PPI among the networks involved in the responses to the five viral infections

The PPI analysis highlighted the genes SARS-CoV-2 shared with other viral infections that have high interaction activity (**Fig 6**). By selecting high interactive genes, we used an analysis of gene enrichment and PPI to identify more information about the function of these genes. It was clear from the results that the genetic pathways associated with rheumatoid arthritis, the AGE-RAGE signaling pathway, malaria, hepatitis B, and influenza A were of high significance (**Fig 7**). The correlation among the host response to rheumatoid arthritis, malaria and SARS-CoV-2 has been mysterious despite the fact that several rheumatoid arthritis and malaria drugs are available, with some efficacy against SARS-CoV-2 infection [63, 64]. Our results suggest that the link between these diseases and infection with SARS-CoV-2 is more related to PPI interactions. In addition, the PPI network showed that these genes are highly significant across other infectious diseases such as EBOV, MERS-CoV and SARS-CoV.

## Conclusion

We compared five transcriptomic profiles for cell host infection with SARS-CoV-2, EBOV, H1N1, MERS-CoV and SARS-CoV. Our analysis identified several key aspects of the host response to SARS-CoV-2 infection where essential immunity genes and biological pathways could be used for understanding the pathogenesis of SARS-CoV-2 infection. Common and specific genetic factors and pathways have been identified that characterize the immunopathology of SARS-CoV-2 infection. Our research outlined the relationship between EBOV's cellular host response and SARS-CoV-2, where many genes and GO terms are enriched. Genes related to immune regulation, including FGF1 and *FOXO1*, and those associated with extreme inflammation, such as *NRCAM* and *SAA2*, have been closely associated with the cellular response to SARS-CoV-2 infection. In addition, common interleukin family members, in particular IL-8 and IL-6, demonstrated a special relationship with SARS-CoV-2 infection, indicating their key importance. The GO evaluation highlighted pathways for RAGE, miRNA and PLA2 inhibitors, which were first identified in this study as possible pathways highly associated with the host response to SARS-CoV-2 infection. Some of these pathways, such as PLA2 inhibitors, may hold the key for potential drugs to manage SARS-CoV-2 infections. The PPI analysis sheds light on genes with high interaction activity that SARS-CoV-2 shares with other viral infections, where the findings showed that the genetic pathways associated with rheumatoid arthritis, the AGE-RAGE signaling system, malaria, hepatitis B, and influenza A were of high significance. Our work also shows that a combination of different types of experimental methods and parameters have been effective in studying the response of SARS-CoV-2 compared to other viral infections.

## Supporting information

S1 Table The information used in this study. (XLS)

S2 Table The information of DEGs associated with the host response of SARS-CoV-2, EBOV, H1N1, MERS-CoV, and SARS-CoV viral infections. (XLSX)

S3 Table The Venn analysis results of DEGs and GO terms uniquely shared across SARS-CoV-2, EBOV, H1N1, MERS-CoV, and SARS-CoV viral infections. (XLSX)

S4 Table Selected gene enrichment analysis of uniquely shared groups of genes across the host response of SARS-CoV-2, EBOV, H1N1, MERS-CoV, and SARS-CoV viral infections. (XLS)

S5 Table The gene expression information of DEGs that SARS-CoV-2 shares with the studied infectious diseases. (XLS)

S6 Table Selected gene enrichment analysis of uniquely shared groups of GO terms across the host response of SARS-CoV-2 and the studied viral infections. (XLS)

S1 Fig The PPI network and gene enrichment analysis of the 173 genes that characterized the host response of SARS-CoV-2. Each node represents a protein and each edge stands for an interaction, colour-coded by the type of evidence.
(TIF)

S2 Fig The PPI network and gene enrichment analysis of the 58 genes that are uniquely shared between COVID-19 and EBOV viral infections. Each node represents a protein and each edge stands for an interaction, colour-coded by the type of evidence.
(TIF)

S3 Fig The PPI network and gene enrichment analysis of the 51 genes that are uniquely shared between SARS-CoV-2 and MERS-CoV viral infections. Each node represents a protein and each edge stands for an interaction, colour-coded by the type of evidence.
(TIF)

S4 Fig The PPI network and gene enrichment analysis of the 31 genes that are uniquely shared among the SARS-CoV-2, EBOV, and MERS-CoV viral infections. Each node represents a protein and each edge stands for an interaction, colour-coded by the type of evidence.
(TIF)

S5 Fig The gene expression heatmap of genes SARS-CoV-2 shares with different viral infections. (TIF)

S6 Fig The PPI network and gene enrichment analysis of genes that are differentially expressed across the studied viral infections and shared with SARS-CoV-2. Each node represents a protein and each edge stands for an interaction, colour-coded by the type of evidence.
(TIF)
