# Supplementary figures and images for: The transcriptomic profiling of SARS-CoV-2 compared to SARS, MERS, EBOV, and H1N1
Source: PLoS One. 2020 Dec 10;15(12):e0243270. doi: 10.1371/journal.pone.0243270 (PMC7728291; doi:10.1371/journal.pone.0243270)

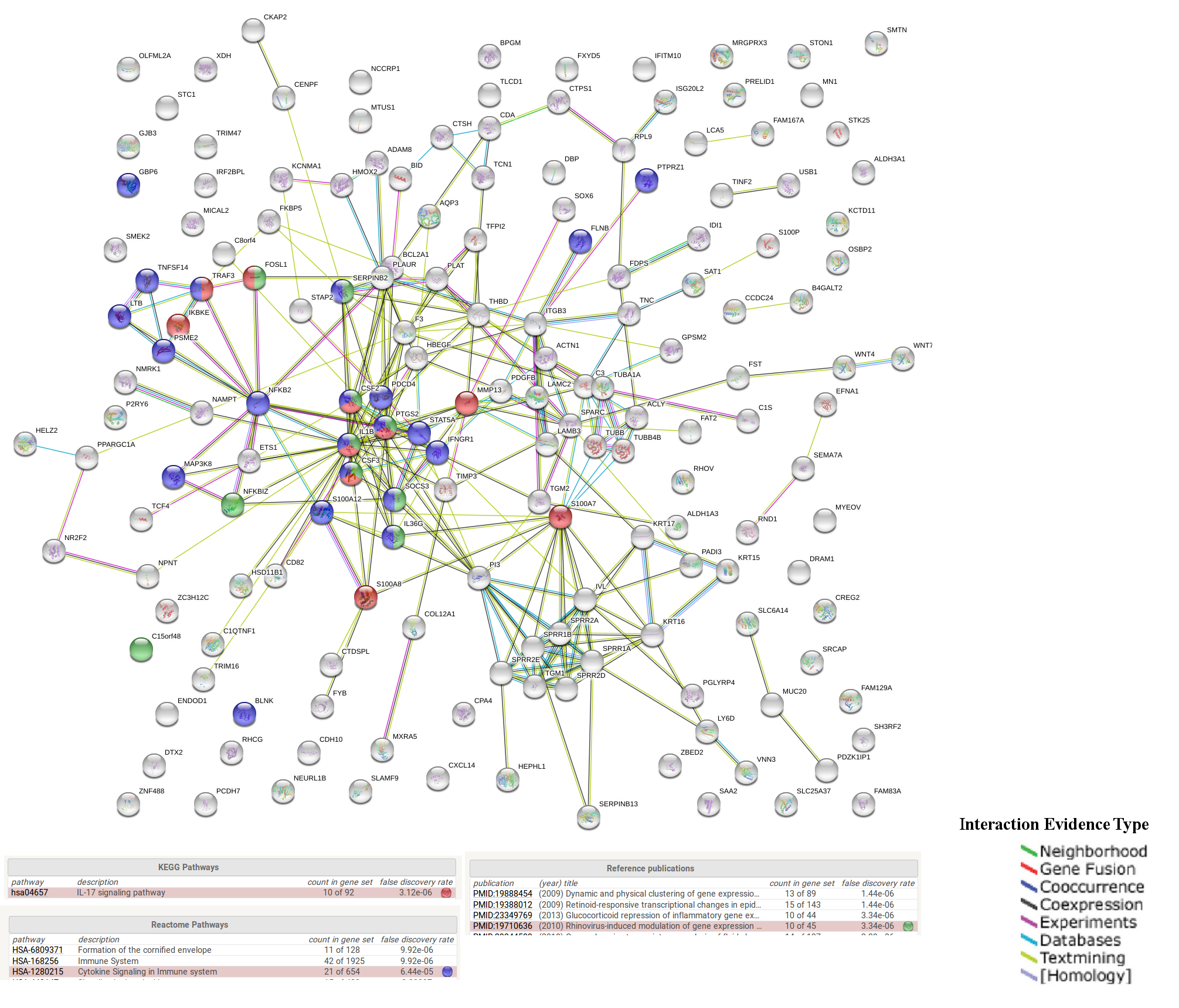

Supplement: S1 Fig — Each node represents a protein and each edge stands for an interaction, colour-coded by the type of evidence. (TIF) [file pone.0243270.s007.tif]

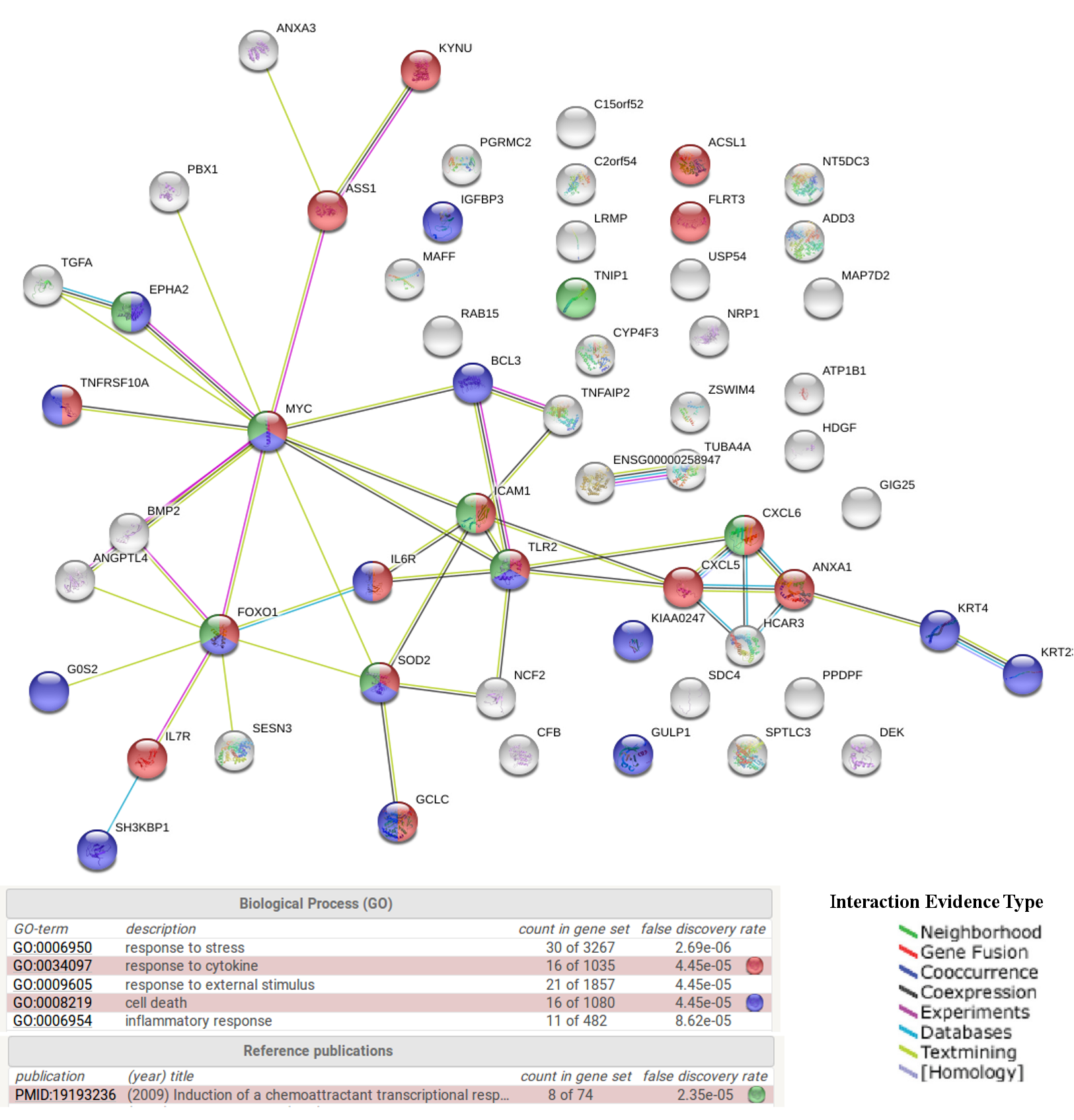

Supplement: S2 Fig — Each node represents a protein and each edge stands for an interaction, colour-coded by the type of evidence. (TIF) [file pone.0243270.s008.tif]

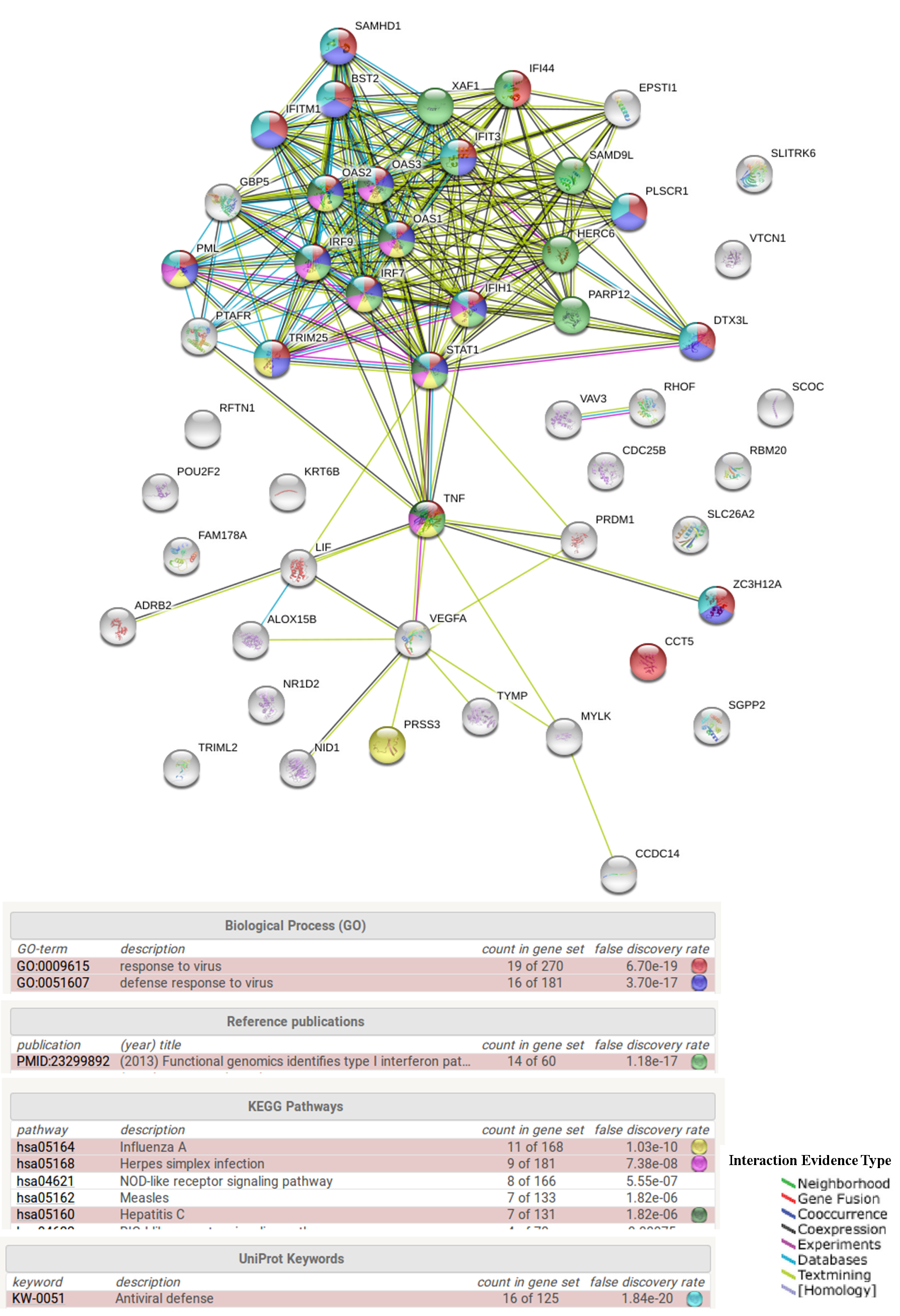

Supplement: S3 Fig — Each node represents a protein and each edge stands for an interaction, colour-coded by the type of evidence. (TIF) [file pone.0243270.s009.tif]

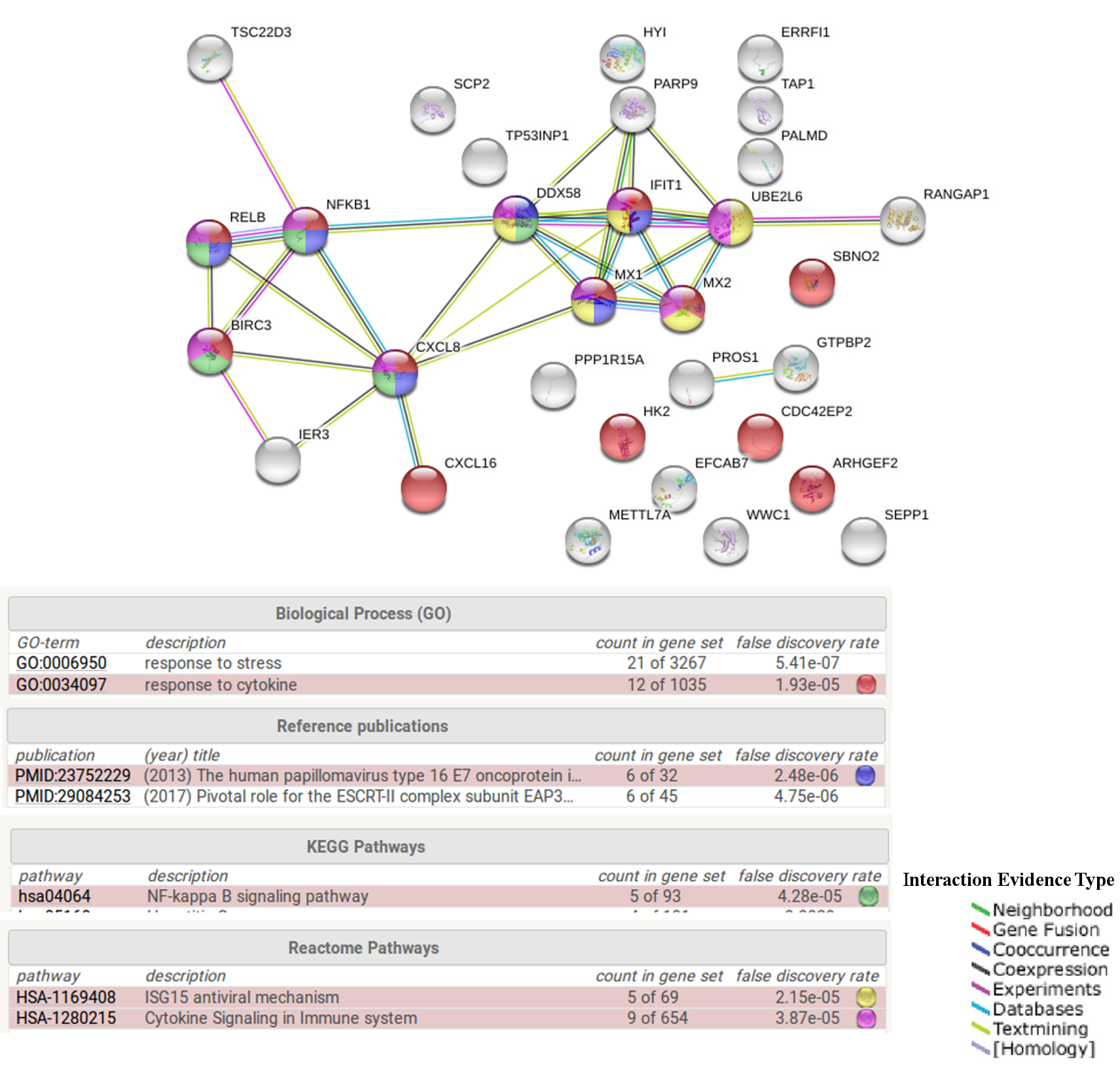

Supplement: S4 Fig — Each node represents a protein and each edge stands for an interaction, colour-coded by the type of evidence. (TIF) [file pone.0243270.s010.tif]

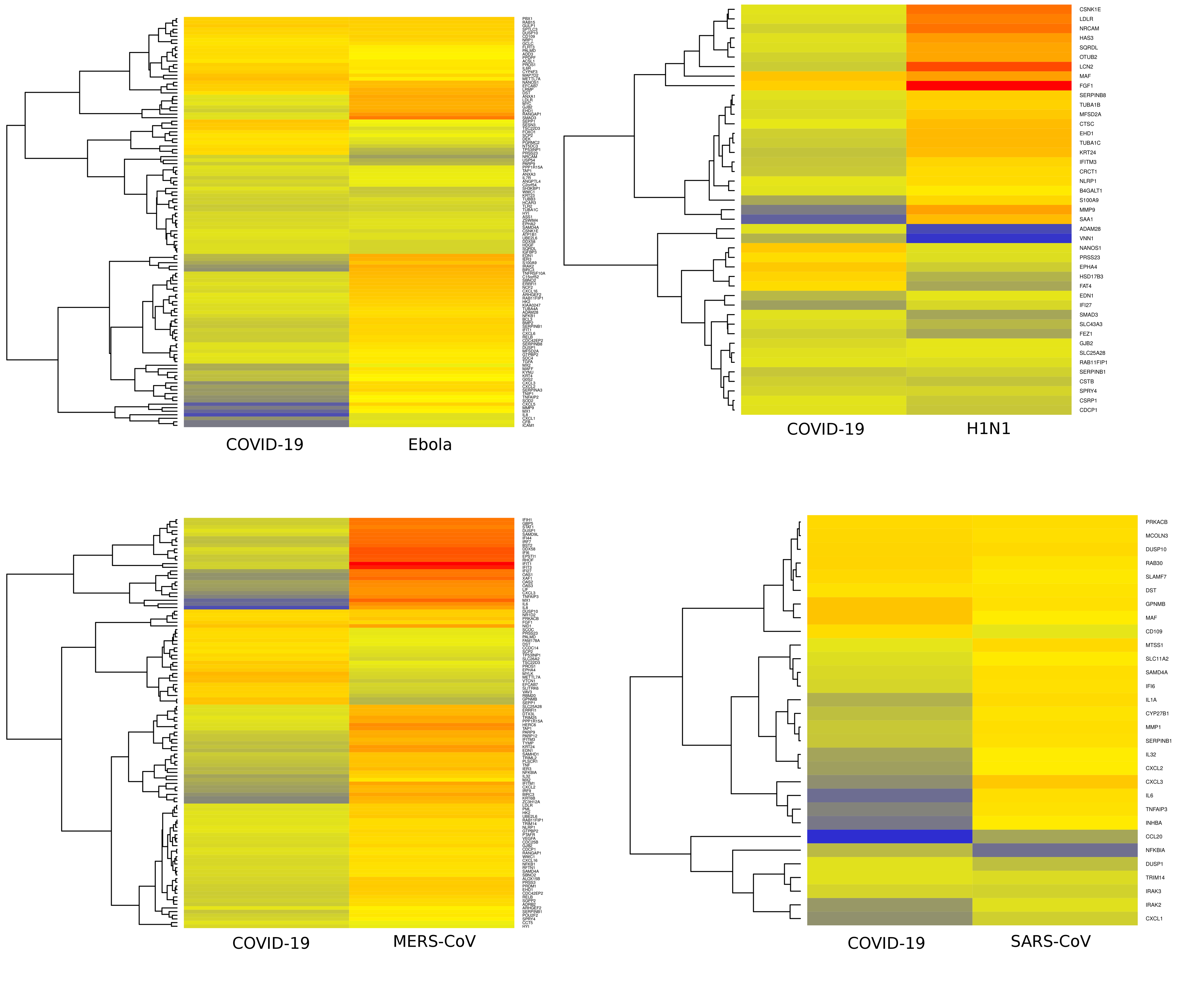

Supplement: S5 Fig — (TIF) [file pone.0243270.s011.tif]

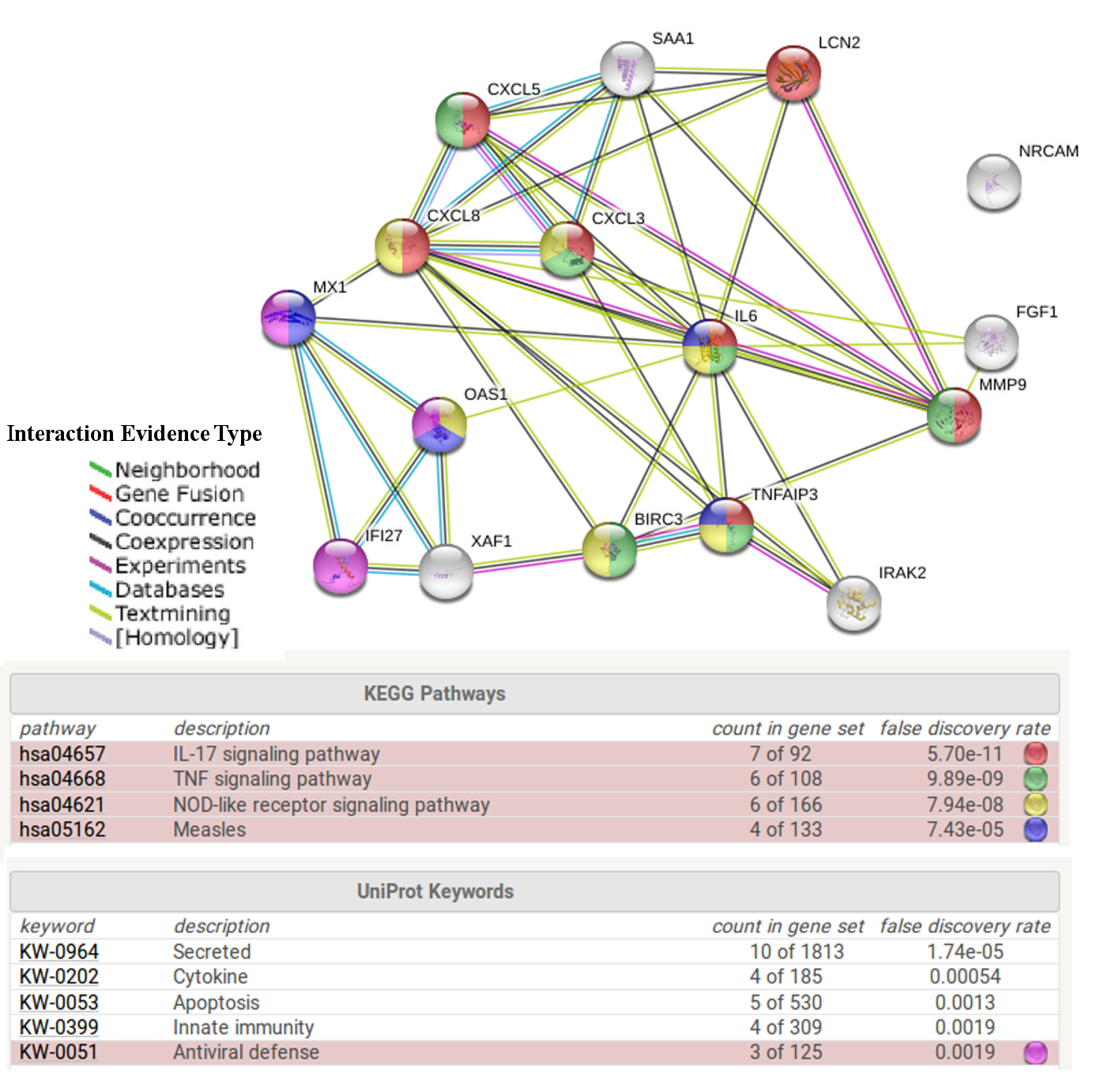

Supplement: S6 Fig — Each node represents a protein and each edge stands for an interaction, colour-coded by the type of evidence. (TIF) [file pone.0243270.s012.tif]
